# Dual Ligand Insertion in gB and gD of Oncolytic Herpes Simplex Viruses for Retargeting to a Producer Vero Cell Line and to Cancer Cells

**DOI:** 10.1128/JVI.02122-17

**Published:** 2018-02-26

**Authors:** Biljana Petrovic, Valerio Leoni, Valentina Gatta, Anna Zaghini, Andrea Vannini, Gabriella Campadelli-Fiume

**Affiliations:** a Department of Experimental, Diagnostic and Specialty Medicine, University of Bologna, Bologna, Italy; b Department of Veterinary Medical Sciences, University of Bologna, Bologna, Italy; University of California, Irvine

**Keywords:** HER2, HSV, retargeting, gD, gB, Vero, oncolytic virus

## Abstract

Oncolytic viruses gain cancer specificity in several ways. Like the majority of viruses, they grow better in cancer cells that are defective in mounting the host response to viruses. Often, they are attenuated by deletion or mutation of virulence genes that counteract the host response or are naturally occurring oncolytic mutants. In contrast, retargeted viruses are not attenuated or deleted; their cancer specificity rests on a modified, specific tropism for cancer receptors. For herpes simplex virus (HSV)-based oncolytics, the detargeting-retargeting strategies employed so far were based on genetic modifications of gD. Recently, we showed that even gH or gB can serve as retargeting tools. To enable the growth of retargeted HSVs in cells that can be used for clinical-grade virus production, a double-retargeting strategy has been developed. Here we show that several sites in the N terminus of gB are suitable to harbor the 20-amino-acid (aa)-long GCN4 peptide, which readdresses HSV tropism to Vero cells expressing the artificial GCN4 receptor and thus enables virus cultivation in the producer noncancer Vero-GCN4R cell line. The gB modifications can be combined with a minimal detargeting modification in gD, consisting in the deletion of two residues, aa 30 and 38, and replacement of aa 38 with the scFv to human epidermal growth factor receptor 2 (HER2), for retargeting to the cancer receptor. The panel of recombinants was analyzed comparatively in terms of virus growth, cell-to-cell spread, cytotoxicity, and *in vivo* antitumor efficacy to define the best double-retargeting strategy.

**IMPORTANCE** There is increasing interest in oncolytic viruses, following FDA and the European Medicines Agency (EMA) approval of HSV Oncovex^GM-CSF^, and, mainly, because they greatly boost the immune response to the tumor and can be combined with immunotherapeutic agents, particularly checkpoint inhibitors. A strategy to gain cancer specificity and avoid virus attenuation is to retarget the virus tropism to cancer-specific receptors of choice. Cultivation of fully retargeted viruses is challenging, since they require cells that express the cancer receptor. We devised a strategy for their cultivation in producer noncancer Vero cell derivatives. Here, we developed a double-retargeting strategy, based on insertion of one ligand in gB for retargeting to a Vero cell derivative and of anti-HER2 ligand in gD for cancer retargeting. These modifications were combined with a minimally destructive detargeting strategy. This study and its companion paper explain the clinical-grade cultivation of retargeted oncolytic HSVs and promote their translation to the clinic.

## INTRODUCTION

Oncolytic viruses constitute a recent class of anticancer therapeutics, which can be armed with cytokines and can be administered in combination with checkpoint inhibitors (1–8). Oncolytic viruses may be wild-type (wt) viruses, natural mutants, animal viruses with tropism for human cells, or genetically engineered viruses. They share the ability to infect, replicate in, and kill cancer cells. Numerous oncolytic viruses from different viral families are being evaluated in clinical trials (9–12). The oncolytic virus originally named Oncovex^GM-CSF^ (where GM-CSF is granulocyte-macrophage colony-stimulating factor [GM-CSF]) has been approved by the FDA and the European Medicines Agency (EMA) against metastatic melanoma (13, 14).

A key requirement for oncolytic viruses is cancer specificity. For a number of viruses, the specificity rests on a higher ability to replicate in cancer cells, which are usually defective in some branches of the innate response (2, 15). Other viruses, exemplified by Oncovex^GM-CSF^, were engineered so as to attenuate them, i.e., to delete virulence genes that counteract the host response (13, 16). Hence, they are defective in replication in noncancer cells and replicate in cancer cells to various degrees. The most highly attenuated viruses may exhibit limited replication even in cancer cells (17).

An alternative strategy to attenuation is tropism retargeting, whereby the viral tropism is retargeted to cancer-specific receptors of choice and detargeted from natural receptors; the viruses are otherwise wt, i.e., nonattenuated (18–22). In our laboratory, we selected as the target receptor human epidermal growth factor receptor 2 (HER2) (20, 22–27), a member of the epidermal growth factor receptor (EGFR) family of receptors, present in a subset of breast, ovary, stomach, and lung cancers. The patients carrying HER2-positive tumors are treated with the anti-HER2 humanized antibodies trastuzumab and/or pertuzumab (28, 29). However, a fraction of patients do not respond (30). Those who respond develop resistance, frequently within a year of treatment, with mechanisms that do not involve the loss of the HER2 ectodomain. In our earlier studies, the tropism retargeting has been achieved by deletion of gD sequences critical for interaction with the gD natural receptors herpesvirus entry mediator (HVEM) and nectin1 (detargeting), either amino acids (aa) 6 to 38 or aa 61 to 218, and their replacement with a single-chain variable-fragment antibody (scFv) to HER2 derived from trastuzumab (20, 22, 31). These recombinants, named R-LM113 and R-LM249, exhibit strong oncolytic/therapeutic activity in nude mice xenotransplanted with human HER2-positive ovary or breast cancers, including metastatic cancers and a glioblastoma model (23–26).

The HER2-retargeted oncolytic HSVs (oHSVs) employed so far in preclinical studies were cultivated in cancer cells, a procedure that may not be approvable for the growth of clinical-grade viruses. Recently, we have developed a strategy, based on double retargeting, for cultivation of retargeted oHSVs in noncancer cells (32). Briefly, gD carries the scFv to HER2 in place of aa 6 to 38 for cancer retargeting, while gH carries the 20-aa-long GCN4 peptide, derived from a yeast transcription factor (recombinant R-213). The GCN4 peptide enabled infection of Vero cells expressing an artificial receptor, named GCN4R, made of an scFv to GCN4 (33) fused to domains II, III, TM, and C tail of nectin1 (32). Subsequently, we showed that also gB can be a tool for retargeting and accepts the insertion of the scFv to HER2 at a specific position, between aa 43 and 44 (34). The choice of Vero cells as recipients of GCN4R rested on the notion that wt Vero cells have been approved by FDA for the clinical-grade preparations of Oncovex^GM-CSF^ (commercial name Imlygic), the derivative named Vero-His (35) is approved for clinical-grade preparations of oncolytic measles viruses, and more generally, wt Vero cells are approved for growth of a number of human vaccines.

The aims of this work were 2-fold: first, to ascertain whether the simultaneous retargeting to two targets, GCN4R and HER2, could be achieved by insertion of the GCN4 peptide in gB and detargeting plus HER2-retargeting via gD; second, to develop a novel, minimally invasive strategy for detargeting gD from its natural receptors. We report that gB can accept the GCN4 retargeting peptide at several positions for *in vitro* cultivation in noncancer cells; one such modification was combined with a gD detargeting strategy based on the deletion of two single amino acids (residues 30 and 38) and replacement of aa 38 with the scFv to HER2 for retargeting to the cancer receptor.

## RESULTS

### Insertion of ligands in gB and in gD for the simultaneous retargeting to two different targets.

**FIG 1:**
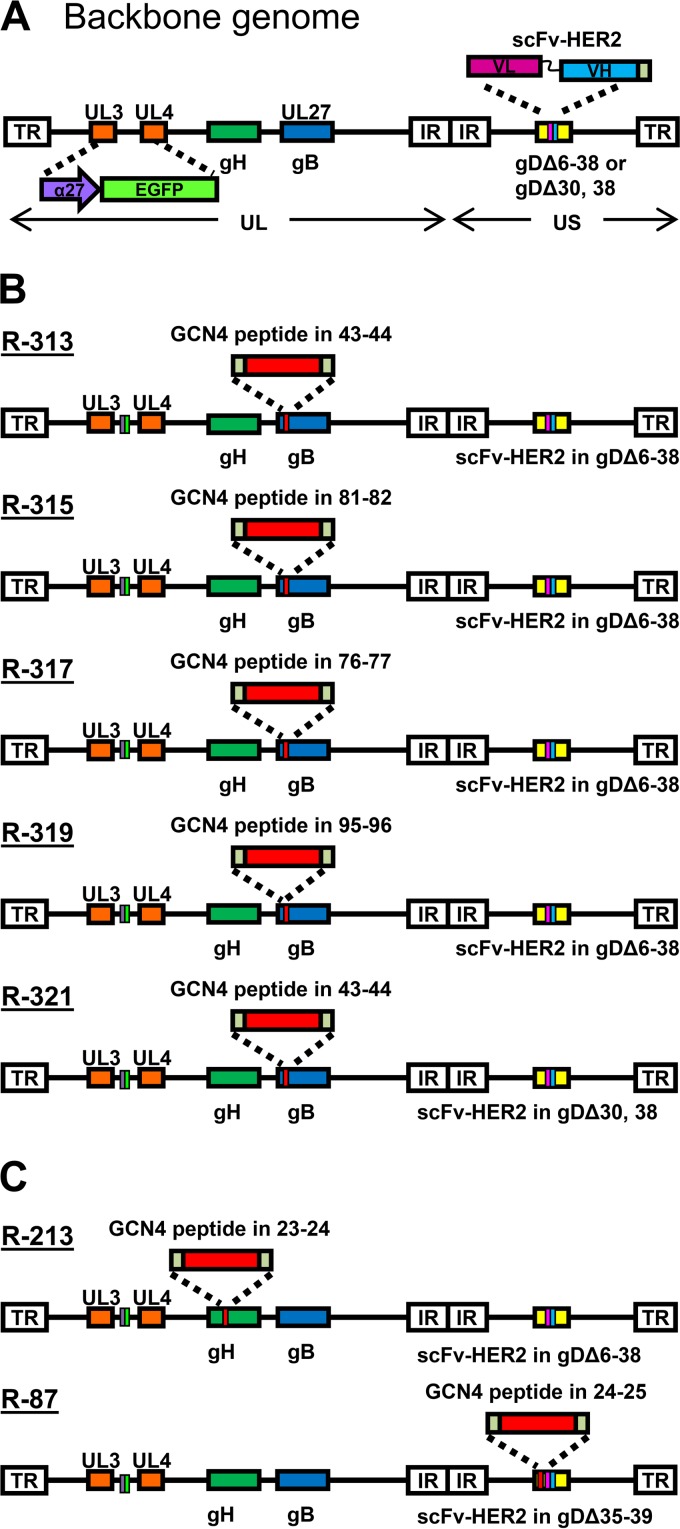
Genome arrangement of recombinants generated in this study. (A) Prototypic genome arrangement of recombinants. Each recombinant carries the BAC sequence and the α27 promoter-driven EGFP (enhanced green fluorescence protein), bracketed by LoxP sites, cloned in the UL3 and UL4 intergenic region and the scFv to HER2 in appropriate sites of gD as detailed below. The unique long (UL) and unique short (US) portions of the genome, bracketed by terminal (TR) and internal (IR) repeats, along with the location of gB and gH genes, are shown. (B) Specific genotypic modifications of gB and gD genes in each recombinant. (C) Specific genotypic modifications in the gH and gD genes of each recombinant.

**FIG 2:**
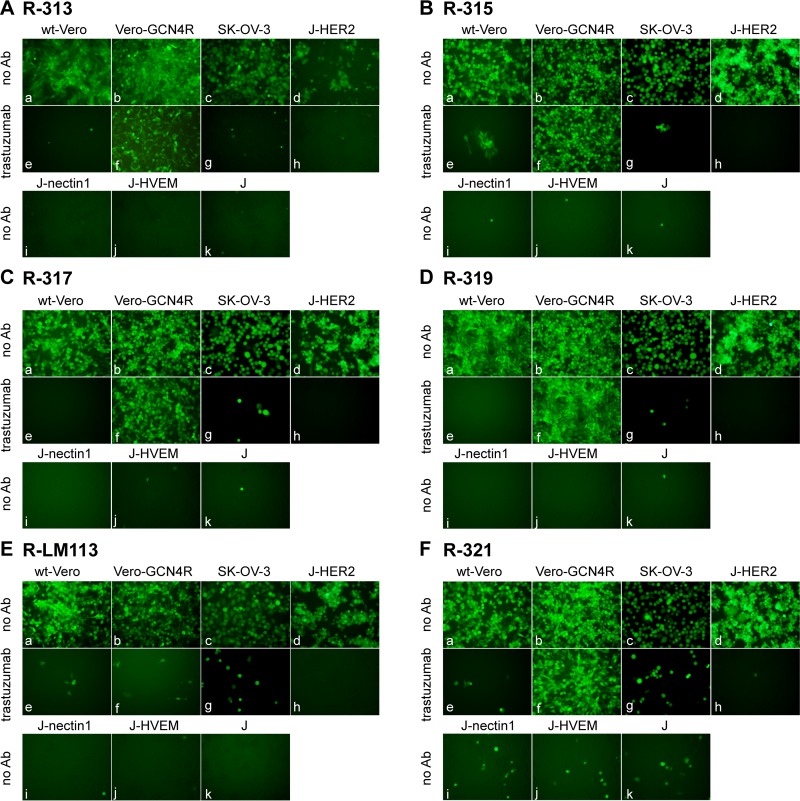
Tropism of R-313, R-315, R-317, R-319, and R-321 recombinants and, for comparison, of R-LM113 in the indicated cell lines. (A to F) The indicated cells were infected with R-313 (A), R-315 (B), R-317 (C), R-319 (D), R-321 (F), and for comparison, R-LM113 (E) at an MOI of 3 PFU/cell and monitored for EGFP expression by fluorescence microscopy 24 h postinfection. J cells express no receptor for wt HSV; J-HER2, J-nectin1, and J-HVEM express the indicated receptor. Infection was carried out in the absence of antibodies (no Ab) or in the presence of the humanized anti-HER2 monoclonal antibody trastuzumab at a concentration of 28 μg/ml. The respective adjustments for level, brightness, and contrast of each subpanel were done as follows: for R-313, a, b, e, +35 +50 +100; c, g, +35 0 +100; d, h, i, j +35 0 0; f, +0/+95 +95; k +35 +75 +100; for R-315, a, b, e, f, +35 +50 +100; c, +35 0 +100; d, h, i, j, k, +35 0 0; g, +35 0 +100; for R-317, a, b, e, f, +35 +50 +100; c, +35 0 +100; d, h, i, j, k, +35 0 0; g, +35 0 +100; for R-319, a, b, e, f, +35 +50 +100; c, +35 0 +100; d, h, i, j, k, +35 0 0; g, +35 0 +100; for R-LM113, a, b, +75 +50 +100; c, +35 0 +180; d, i, j, k, +35 0 0; e, f, +35 +50 +100; g, +35 0 +100; h, +35 −150 0; for R-321, a, b, e, f, +35 +50 +100; c, +35 0 +100; d, h, i, j, k, +35 0 0; g, +35 0 +100.

We generated four recombinants, R-313, R-315, R-317, and R-319, carrying the GCN4 peptide in gB at one of four sites, i.e., between aa 43 and 44, 81 and 82, 76 and 77, and 95 and 96, and carrying the scFv to HER2 in gD, in place of aa 6 to 38 (Fig. 1 and Table 1). A description of these viruses is given in European patent application PCT/EP2017/063944 (M. G. Campadelli and B. Petrovic, 14 December 2017). The tropism of the recombinants was evaluated in the HER2-positive SK-OV-3 cancer cells, in the Vero-GCN4R, in wt Vero cells, and in derivatives of the receptor-negative J cells, transgenically expressing a single receptor, e.g., HER2, nectin1, or HVEM (20, 36). R-LM113, retargeted to HER2 but not to GCN4R, was included as a control. Figure 2A to D shows that the recombinant R-313, R-315, R-317, and R-319 viruses were retargeted to GCN4R, as indicated by the ability to infect Vero-GCN4R cells, in the presence of the anti-HER2 monoclonal antibody (MAb) trastuzumab. All recombinants were retargeted to HER2, as indicated by ability to infect J-HER2 and SK-OV-3 cells in a trastuzumab-dependent fashion. This property is shared with R-LM113 (Fig. 2E). Consistent with the deletion of aa 6 to 38 (Δ6–38) in gD and replacement of the deleted sequences with the scFv to HER2 (22), all recombinants failed to infect J-HVEM and J-nectin1 cells, i.e., they were detargeted from natural gD receptors. They infected the wt Vero cells in a trastuzumab-inhibited fashion, very likely through the simian orthologue of HER2. Indeed, the whole-genome sequence of Vero cells is incomplete, and so far, there is no documentation of a HER2 homologue in this cell line. Nonetheless, Vero cells were isolated from an African green monkey (Chlorocebus sp.), and the sequence of the Chlorocebus genome contains the HER2 homologue (Chlorocebus sabaeus; RefSeq accession number XM_008012845.1) with 98% identity with the human HER2. We conclude from these results that the four insertion sites tested were all suitable for insertion in that the generated recombinants were viable, implying that gB carried out the fusogenic function. More importantly, the inserted GCN4 peptide mediated infection through the GCN4R, i.e., it was suitably located in gB not only to prevent any detrimental effect on gB function but also to contribute to its entry function, i.e., to the GCN4R-dependent infection and consequent gB activation. It was possible to combine the insertions in gB with the retargeting to HER2 through deletion/scFv insertion in gD.

**TABLE 1:** Major genotypic and phenotypic properties of recombinants described in this study

Recombinant	GCN4 position in gB	scFv-HER2 position in gD	GCN4 position in gH	Retargeting to HER2	Detargeting from nectin1/HVEM	Reference or source
R-313	43–44	Δ6–38	None	+	+	This paper
R-315	81–82	Δ6–38	None	+	+	This paper
R-317	76–77	Δ6–38	None	+	+	This paper
R-319	95–96	Δ6–38	None	+	+	This paper
R-321	43–44	Δ30, Δ38	None	+	+	This paper
R-87	None	Δ35–39 plus GCN4 between aa 24 and 25	None	+	+	37
R-213	None	Δ6–38	23–24	+	+	32
R-LM113	None	Δ6–38	None	+	+	22
R-LM5	None	No scFv, no deletion	None	−	−	22

### Novel gD detargeting strategy.

In the four recombinants described above, the detargeting from the natural gD receptors was achieved by deletion of the aa 6 to 38 region, which contains residues critical for interaction with HVEM and nectin1, and its replacement with the scFv to HER2. Here we asked whether the detargeting could be achieved by a less invasive strategy. We deleted aa 30 and 38 and replaced aa 38 with the scFv to HER2. The modification in gB was the same as the one present in R-313, i.e., the insertion of the GCN4 peptide between aa 43 and 44. The resulting recombinant was named R-321 (Table 1; for a schematic drawing of the genotype, see Fig. 1B). Its tropism is shown in Fig. 2F. R-321 failed to infect J-nectin1 and J-HVEM cells. Thus, the simple deletion of the two residues in gD was sufficient to detarget the virus tropism from the two natural gD receptors. R-321 was retargeted to HER2 (trastuzumab-dependent infection of SK-OV-3 and J-HER2 cells); hence, the insertion of the scFv in place of aa 38 led to retargeting. The retargeting via the GCN4 insertion in gB was not modified relative to that seen in R-313, as expected.

### Replication and cell-to-cell spread of the double-retargeted recombinants.

**FIG 3:**
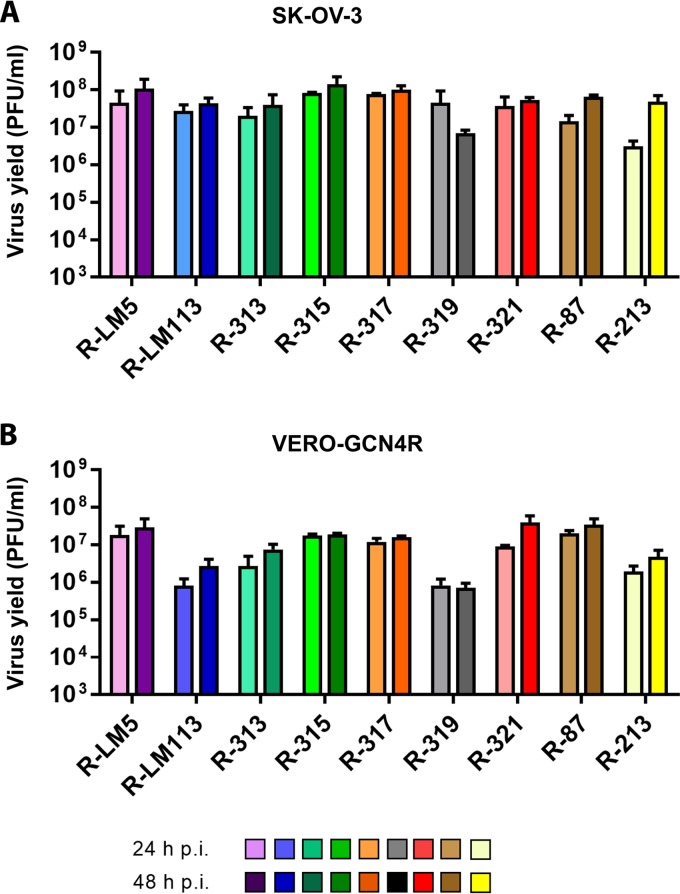
Yields of R-313, R-315, R-317, R-319, and R-321 recombinants and of R-LM5, R-LM113, R-213, and R-87 for comparison. (A, B) SK-OV-3 (A) and Vero-GCN4R (B) cells were infected with the indicated virus recombinants at 0.1 PFU/cell. Progeny virus was titrated in SK-OV-3 cells at 24 or 48 h after infection. Results represent the averages ± SD from triplicates.

We measured the growth capacity of the recombinants in SK-OV-3 and in Vero-GCN4R cells. Figure 3A and B shows results of a typical experiment. Three of the recombinants, R-315, R-317, and R-321, could not be differentiated one from the other and exhibited a high replication capacity in SK-OV-3 cells at 48 h. They replicated about as well as R-LM113, which carries only the aa 6 to 38 deletion and the scFv insertion in place of the deleted sequence and has no modification in gB. The recombinant replicated as efficiently as R-LM5, which carries no deletion and no retargeting moiety at all (22). With respect to R-LM113, we note that this recombinant replicated for one passage in wt Vero cells and their Vero-GCN4R derivative; however, numerous efforts to passage serially R-LM113 in these cells were unsuccessful and did not yield any progeny. Whether the defect in serial passages depends on low density of the receptor, low affinity-avidity between simian HER2 and the scFv to human HER2 inserted in R-LM113, or another factor, remains to be investigated. In both cells, R-315, R-317, and R-321 replicated better that R-313 and R-319. In Vero-GCN4R, the yield of all recombinants was about 0.5 to 1 log lower than that in SK-OV-3 cells, in agreement with earlier observations on gH-retargeted recombinants (32). We conclude from these results that not all the insertion sites in gB are equivalent with respect to virus infection/replication ability.

**FIG 4:**
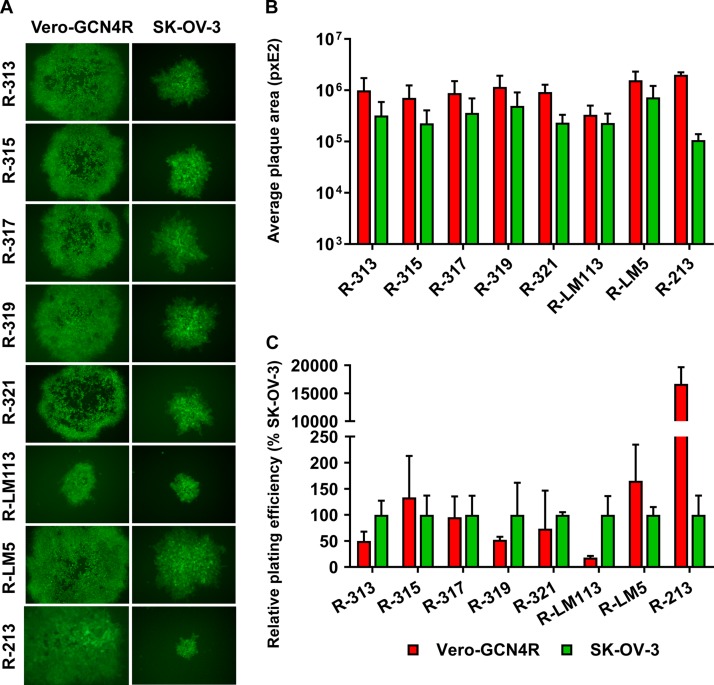
Plating efficiency and relative plaque sizes of the indicated recombinants in Vero-GCN4R and SK-OV-3. (A) A typical plaque is shown for each recombinant in the indicated cells. (B) Average plaque size of the indicated recombinants in Vero-GCN4R and SK-OV-3. Six pictures were taken for each recombinant. Plaque areas were measured by means of Nis Elements-Imaging software (Nikon). (C) Replicate aliquots of recombinants were plated in SK-OV-3 and Vero-GCN4R cells. Plaques were scored 3 days later. The relative number of plaques formed by each virus in the indicated cell line is reported as percentage of the number of plaques formed in SK-OV-3 cells. Results represent the averages ± SD from triplicates. The level, brightness, and contrast of the panel were adjusted as follows: +30 +80 +30. The level, brightness, and contrast of R-213 pictures were adjusted as follows: 0 +100 +30.

Figure 3A and B also shows the replication of R-87, for comparison. R-87 is described in a companion paper (37). It carries the same ligands as the recombinants described in this paper, i.e., the scFv to HER2 and the GCN4 peptide. However, both ligands are engineered in gD. In particular, the scFv to HER2 replaces aa 35 to 39, and the GCN4 is inserted between aa 24 and 25 (Fig. 1C and Table 1). Figure 3A and B shows that the yields of R-87 were very similar to those of the best-performing recombinants, R-315, R-317, and R-321, notwithstanding the differences in the design of the two sets of viruses. A comparison was made also to R-213, a recombinant that carries the GCN4 peptide in gH, between aa 23 and 24, and the same modifications in gD as R-313, R-315, R-317, and R-319 (Fig. 1B) (32). Overall, R-213 replicated to yields similar to those of the recombinants generated in this study in SK-OV-3 cells at 48 h and at a somewhat lower yield in Vero-GCN4R cells (Fig. 3). Next, we measured the cell-to-cell spread. Typical examples of plaques are shown in Fig. 4A, and average plaque sizes are quantified in Fig. 4B. All recombinants produced medium-to-large plaques in Vero-GCN4R cells and medium-sized plaques in SK-OV-3 cells. Importantly, all recombinants were more effective in cell-to-cell spread in Vero-GCN4R cells than R-LM113 (Fig. 4B). With respect to the relative number of plaques in Vero-GCN4R and in SK-OV-3 cells, there was no significant difference among R-313, R.315, R-317, and R-319 (Fig. 4C). There was a clear advantage of the recombinants over R-LM113 in Vero-GCN4R cells, as expected. With respect to plaque number, but not to plaque size, the recombinant R-213, which carries the GCN4 in gH (32), was superior in Vero-GCN4R cells (Fig. 4C). Thus, although the gH recombinant needs further improvements, it shows interesting properties worth to be explored.

### Cytotoxicity induced by the double-retargeted recombinants.

**FIG 5:**
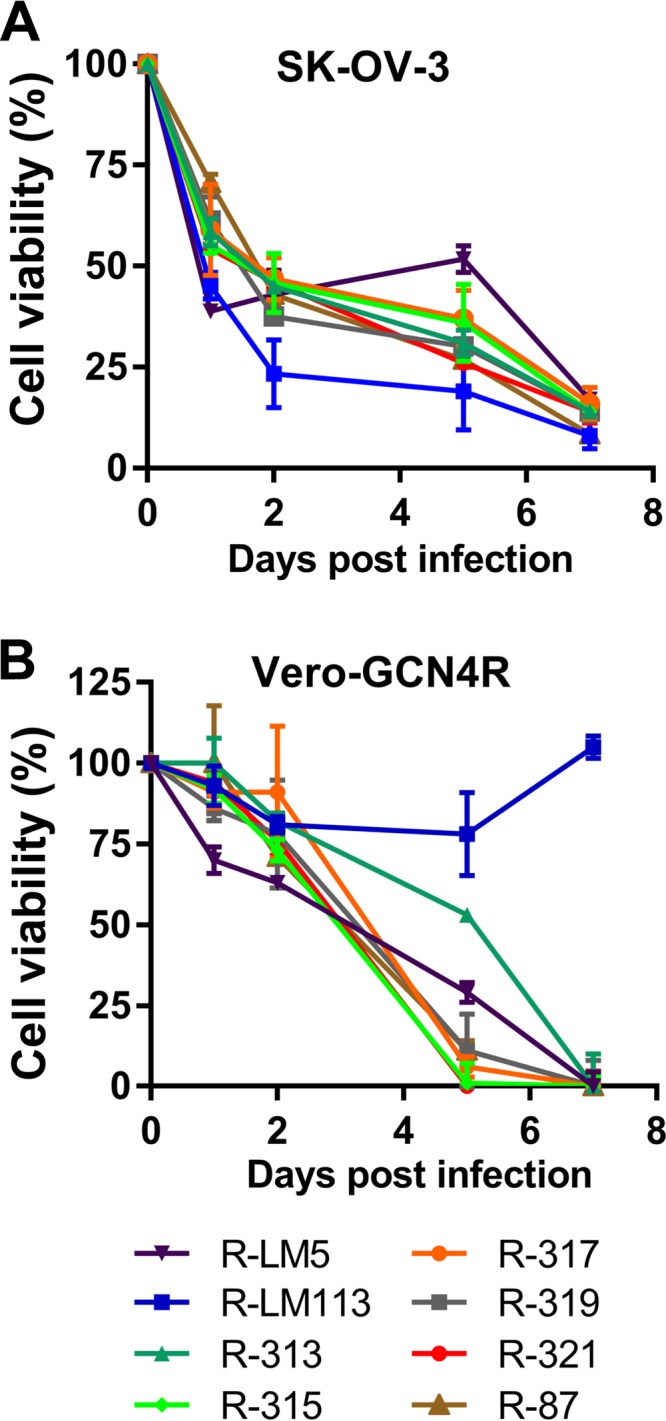
Killing ability of the indicated recombinants for SK-OV-3 and Vero-GCN4R cells. (A, B) SK-OV-3 (A) or Vero-GCN4R (B) cells were infected with the indicated recombinants or with R-LM5 and R-LM113 as controls, at 3 PFU/cell (Vero-GCN4R) or 10 PFU/cell (SK-OV-3). Cell viability was quantified by alamarBlue assay at the indicated days after infection. Results represent a typical experiment; each sample is the average ± SD from triplicate assays.

An important property for any candidate oncolytic virus is the ability to kill cells. Hence, it was critical to ascertain whether the GCN4 retargeting via gB affected the virus-induced cytotoxicity. Monolayers of SK-OV-3 and Vero-GCN4R cells were infected with the recombinants, with R-87 for comparison and with R-LM5 and R-LM113 as controls. Cytotoxicity was measured by means of alamarBlue at the indicated days after infection. Figure 5 shows that all recombinants exerted similar cytotoxic effects. The exception was R-LM113 in Vero-GCN4R cells, as expected.

### Oncolytic efficacy of the double-retargeted recombinant R-317 in immunocompetent mice.

**FIG 6:**
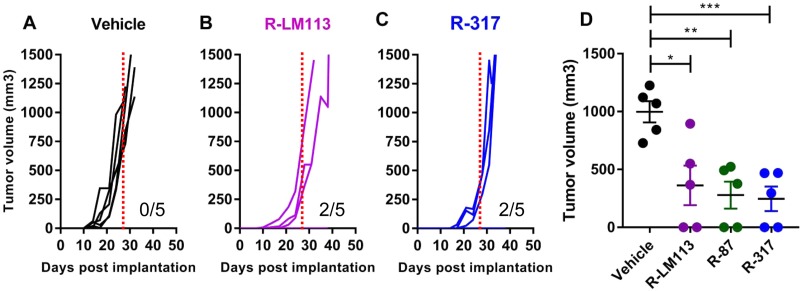
Antitumor activity of R-317. (A to C) Groups of 5 mice from the hHER2-transgenic C56BL6 strain were implanted with hHER2-LLC-1 cells in the left flank. Starting 3 days later, mice received four intratumoral treatments (1 × 10E8 PFU/treatment) at 3 to 4 days' intervals with R-317 alone and with R-LM113 and R-87 as controls. Tumor volumes and the number of tumor-free animals for each treatment group are shown. (D) Distribution of the tumor size at 28 days after the initial treatment. Statistical significance was calculated using the *t* test: *, *P* < 0.05; **, *P* < 0.01; ***, *P* < 0.001. This experiment is the same as that shown in Fig. 7 of the companion paper (37).

We selected R-317, one of the best-performing double-retargeted recombinants, to evaluate the oncolytic efficacy in immunocompetent mice. The animal model will be described elsewhere in detail under different coauthorship (V. Leoni, A. Vannini, V. Gatta, J. Rambaldi, M. Sanapo, C. Barboni, A. Zaghini, P. Nanni, P.-L. Lollini, C. Casiraghi, G. Campadelli-Fiume, unpublished data). Essentially, it consists of the Lewis lung murine carcinoma 1 (LLC-1) cells made transgenic for human HER2 (hHER2-LLC-1). The cancer cells were implanted in a strain of the syngeneic C57BL/6 mice, which are transgenic for, and hence tolerant to, hHER2. Three days after implantation of the tumor cells, R-317 was administered perintratumorally (i.t.) at 3 to 4 days' intervals, with 1 × 10E8 PFU/each injection, for a total of 4 treatments. To enable comparison, we included in the experiment the prototypic R-LM113 and R-87 described in the companion paper (37). Figure 6A to C shows that the antitumor efficacy of R-317 was very similar to those of R-LM113 and of R-87. The tumor size at 28 days was significantly different from that in untreated mice (Fig. 6D).

## DISCUSSION

gB is a highly structured glycoprotein, little prone to accept insertions or mutations, except for the N-terminal region up to about aa 100. The N-terminal region is highly flexible and was disordered in the gB postfusion crystal structure (38–42). Previously, Potel et al. inserted the green fluorescent protein (GFP) moiety in gB at residues 43 to 44; the chimeric form of gB gave rise to a viable recombinant, indicating that the fusion-performing activity of gB had not been hampered (43). Gallagher et al. inserted fluorescent proteins in each of the three globular domains of gB. Only one-third of the constructs were functional in the cell-cell fusion assay; in the functional constructs, the inserts were located either in the N terminus, up to residue 100, or at residues 470 and 481 (44). A remarkable difference between those studies and the current one is that in the earlier studies the inserted fluorescent proteins were not employed as novel retargeting ligands. Hence, it was unknown whether retargeting could be achieved by ligand insertion at these sites. In a previous work, we inserted the scFv to HER2 in gB between aa 43 and 44; the viable recombinant indicated that this is an appropriate site for insertion of a retargeting ligand (34). It was unknown whether other insertion sites enabled the generation of viable recombinants and whether the recombinants were retargeted. Recently, we developed a double-retargeting strategy for growth of clinical-grade retargeted oncolytic HSVs. The strategy is based on the simultaneous retargeting to the HER2 cancer receptor (or other cancer targets of choice) and to the GCN4R present in the producer Vero cells (32). The aim of the current work was to optimize two series of genetic modifications, i.e., ligand insertions in gB finalized to *in vitro* growth in noncancer cells, and to define a novel, minimally destructive strategy for detargeting from natural gD receptors and retargeting to cancer receptors. We generated a recombinant carrying the two series of modifications. The novel data to emerge are as follows.

gB can accept the insertion of the GCN4 retargeting peptide at various sites in the N terminus. The investigated sites were not equivalent one to the other. Thus, the highest yields were achieved by R-315 and R-317, which harbor inserts at aa 81 and 82 or aa 76 and 77, respectively. The yields of these recombinants were very similar to that of R-LM113, which does not carry any modification in gB, suggesting that the perturbations to gB induced by the GCN4 peptide at the aa 81 and 82 or aa 76 and 77 sites had a negligible effect. A decrease in virus yield was observed with R-313 and R-319, which carry the GCN4 insert between aa 43 and 44 or between aa 95 and 96. The latter insertion site is close to the downstream region, which does not tolerate mutagenesis (41, 45). With respect to cell-to-cell spread in SK-OV-3 cells, the recombinants R-313, R-315, R-317, and R-319 did not significantly differ one from the other. R-319 exhibited the highest spread capacity in Vero-GCN4R cells. Surprisingly, the recombinants performed somewhat better than the parental R-LM113, with which they share the same gD modifications, hinting that the modifications to gB favor rather than hamper cell-to-cell spread of the virus. All in all, it appears that the added ability to interact with a gB receptor (in this case, GCN4R) adds to the cell-to-cell spread capacity of the recombinants without hampering the virus growth capacity. We note that mutagenesis of gH or gB at some sites resulted in forms of the glycoproteins with enhanced cell-cell fusion activity, interpreted as a promotion of gH or gB “activation” (19, 46–48). R-317, one of the best-performing double recombinants, was also evaluated for *in vivo* antitumor efficacy, in an immunocompetent mouse model. The model will be described elsewhere (Leoni et al., unpublished). It consists of the C57BL/6 mouse transgenic and hence tolerant to human HER2 (hHER2) and the murine Lewis lung carcinoma 1 (LLC-1) cells made transgenic for hHER2. As noted by several groups, murine cells, including cancer cells, are scarcely permissive to HSV-1 (49, 50). The cells syngeneic with the C57BL/6 mice are among the most resistant. As a consequence, the model underestimates the efficacy of oncolytic HSVs. As expected from the cell culture replication, the antitumor efficacy of R-317 was very similar to that of R-LM113 and of R-87. Thus, the *in vitro* comparative properties are predictive of the *in vivo* antitumor efficacy, and a double-retargeted recombinant is as effective as the singly retargeted R-LM113 virus.

### Novel detargeting strategy.

In earlier retargeted oncolytic HSVs, detargeting was a more demanding task than the actual retargeting (18, 20, 51). The reason for that was that the actual location of the nectin1 binding site in gD was not fully known. Taking advantage of the elucidation of the cocrystal structure of nectin1-bound gD (52), here we designed a less invasive detargeting strategy (R-321). It consists of the deletion of two residues, aa 30 and aa 38, structurally involved in the interaction of gD with HVEM and nectin1, respectively (52–54). Our data show that the two single deletions and the replacement of aa 38 with scFv to HER2 were sufficient to detarget the HSV tropism from both natural receptors. Consistent with current data, the single mutagenesis of aa 38 was sufficient for nectin1 detargeting (21). It is worth comparing the growth properties of R-313 and R-321. The two recombinants share the same gB modifications and differ in the portions of gD deleted for detargeting purposes. R-321 grew to about 1-log-higher yield than R-313. Thus, decreasing the deleted portion of gD significantly rescued viral replication. Altogether, the current study extends our notions on gB as a retargeting tool and combines the retargeting via gB to a novel detargeting strategy via gD.

In a companion paper, we show that double retargeting is feasible also by the simultaneous insertion of both the GCN4 peptide and the scFv in gD (37). Even in that case, the optimization can be achieved by a novel gD detargeting strategy. Results on R-87 were included in this study for comparison. Cumulatively, the two strategies—the double gD retargeting and the gB-gD combination retargeting—result in recombinants that replicate at comparable yields. These studies will help move the field of retargeted oncolytic HSVs to the translational phase.

## MATERIALS AND METHODS

### Cells and viruses.

The J cells (negative for HSV receptors) and their derivatives that transgenically express HER2, nectin1, or HVEM were described previously (20, 55). The Vero-GCN4R cells were derived from Vero cells (ATCC CCL-81) as described previously (32). Wild-type (wt) Vero cells were obtained from ATCC. The above-described cells were grown in Dulbecco's modified Eagle medium (DMEM; catalog number 31600-083; Gibco Laboratories) supplemented with 5% fetal bovine serum (FBS) (EU approved, South American origin, catalog number 10270-106; Gibco Laboratories). The SK-OV-3 cells were purchased from ATCC and cultured as recommended by ATCC, i.e., grown in RPMI 1640-Glutamax (catalog number 61870010; Gibco Laboratories) supplemented with 10% heat-inactivated fetal bovine serum. hHER2-LLC-1 cells are Lewis lung murine carcinoma 1 (LLC-1) cells purchased from ATCC and made transgenic for human HER2. This transgenic cell line will be described elsewhere in detail under different coauthorship (Leoni et al., unpublished). The recombinant viruses R-LM5 and R-LM113 were described previously (22).

### Engineering of HSV recombinants expressing genetically modified gBs.

A description of these viruses is given in European patent application PCT/EP2017/063944 (Campadelli and Petrovic, 14 December 2017). First, we engineered R-313 by insertion of the sequence encoding the GCN4 peptide between aa 43 and 44 of immature gB (corresponding to aa 13 and 14 of mature gB after cleavage of the signal sequence, which encompasses aa 1 to 30). The starting genome was the bacterial artificial chromosome (BAC) LM113, which carries scFv-HER2 in place of aa 6 to 38 of gD, LOX-P-bracketed pBeloBAC11, and enhanced GFP (EGFP) sequences inserted between U_L_3 and U_L_4 of the HSV-1 genome (27). The engineering was performed by *galK* recombineering (56). The GalK cassette with homology arms to gB was amplified by means of primers gB43GalKfor and gB43GalKrev (Table 2) using pgalK as the template. This cassette was electroporated in SW102 bacteria carrying the BAC LM113. The recombinant clones carrying the *galK* cassette were selected as described previously (22) and screened by colony PCR by means of oligonucleotides galK_129_f and galK_417_r (Table 3). Next, the GCN4 peptide cassette with the downstream and upstream Ser-Gly linkers, bracketed by homology arms to gB, was generated through the annealing and extension of oligonucleotides GCN4gB_43_44_fB and GCN4gB_43_44_rB (Table 2), which introduce a silent BamHI restriction site, to enable the screening of colonies. The recombinant clones were screened for the presence of GCN4 peptide by colony PCR with primers gB_ext_for and gB_431_rev (Table 3). R-315 carries the insertion of GCN4 peptide between aa 81 and 82 of HSV gB in the HSV recombinant already expressing an scFv-HER2 in the deletion of aa 6 to 38 in gD. R-317 carries the insertion of GCN4 peptide between aa 76 and 77 of HSV gB in the HSV recombinant already expressing an scFv-HER2 in the deletion of aa 6 to 38 in gD. R-319 carries the insertion of GCN4 peptide between aa 95 and 96 of HSV gB in the HSV recombinant already expressing an scFv-HER2 in the deletion of aa 6 to 38 in gD. R-315, R-317, and R-319 were engineered as detailed above for R-313, by means of the oligonucleotides reported in Table 2, and screened by PCR by means of the oligonucleotides reported in Table 3. R-321 was engineered by reintroduction of aa 6 to 29 and 31 to 37 of gD in the HSV recombinant R-313, which carries an scFv-HER2 in the deletion of aa 6 to 38 in gD and the GCN4 peptide between aa 43 and 44 in gB. First, the *galK* cassette was amplified by means of primers gD5_galK_f, TTGTCGTCATAGTGGGCCTCCATGGGGTCCGCGGCAAATATGCCTTGGCGCCTGTTGACAATTAATCATCGGCA, and scFv_galK_rev, GAGGCGGACAGGGAGCTCGGGGACTGGGTCATCTGGATATCGGAATTCTCTCAGCACTGTCCTGCTCCTT using pgalK as the template. Next, the oligonucleotide that comprises aa 6 to 29 and aa 31 to 37 of gD was generated through the annealing and extension of primers gDdel30_38for, TTGTCGTCATAGTGGGCCTCCATGGGGTCCGCGGCAAATATGCCTTGGCGGATGCCTCTCTCAAGATGGCCGACCCCAATCGCTTTCGCGGCAAAGACCTTCCGGTCC, and gDdel30_38rev, GAGGCGGACAGGGAGCTCGGGGACTGGGTCATCTGGATATCGGAATTCTCCACGCGCCGGACCCCCGGAGGGGTCAGCTGGTCCAGGACCGGAAGGTCTTTGCCGCGA.

**TABLE 2:** Oligonucleotides employed to engineer the indicated recombinant genomes

Recombinant	GalK recombination		GCN4 recombination	
	Primer	Sequence	Primer	Sequence
R-313	gB43GalKfor	GGTGGCGTCGGCGGCTCCGAGTTCCCCCGGCACGCCTGGGGTCGCGGCCGCGCCTGTTGACAATTAATCATCGGCA	GCN4gB_43_44_fB	GGTGGCGTCGGCGGCTCCGAGTTCCCCCGGCACGCCTGGGGTCGCGGCCGCGGGATCCAAGAACTACCACCTGGAGAACGAGGTGGCCAGACTGAAGAAGCTGGTGGGCAGC
	gB43GalKrev	GGCCAGGGGCGGGCGGCGCCGGAGTGGCAGGTCCCCCGTTCGCCGCCTGGGTTCAGCACTGTCCTGCTCCTT	GCN4gB_43_44_rB	GGCCAGGGGCGGGCGGCGCCGGAGTGGCAGGTCCCCCGTTCGCCGCCTGGGTGCTGCCCACCAGCTTCTTCAGTCTGGCCACCTCGTTCTCCAGGTGGTAGTTCTTGGATCC
R-315	gB81fGALK	CGGGGGACACGAAACCGAAGAAGAACAAAAAACCGAAAAACCCACCGCCGCCGCCTGTTGACAATTAATCATCGGCA	gB_81_GCN4_for	CGGGGGACACGAAACCGAAGAAGAACAAAAAACCGAAAAACCCACCGCCGCCGGGATCCAAGAACTACCACCTGGAGAACGAGGTGGCCAGACTGAAGAAGCTGGTGGGCAGC
	gB81GALKrev	CGCAGGGTGGCGTGGCCCGCGGCGACGGTCGCGTTGTCGCCGGCGGGGCGTCAGCACTGTCCTGCTCCTT	gB_81_GCN4_rev	CGCAGGGTGGCGTGGCCCGCGGCGACGGTCGCGTTGTCGCCGGCGGGGCGGCTGCCCACCAGCTTCTTCAGTCTGGCCACCTCGTTCTCCAGGTGGTAGTTCTTGGATCC
R-317	gB_76_galK_for	GGCCCCGCCCCAACGGGGGACACGAAACCGAAGAAGAACAAAAAACCGAAACCTGTTGACAATTAATCATCGGCA	gB_76_GCN4_for	GGCCCCGCCCCAACGGGGGACACGAAACCGAAGAAGAACAAAAAACCGAAAGGATCCAAGAACTACCACCTGGAGAACGAGGTGGCCAGACTGAAGAAGCTGGTGGGCAGC
	gB_76_galK_rev	CCCGCGGCGACGGTCGCGTTGTCGCCGGCGGGGCGCGGCGGCGGTGGGTTTCAGCACTGTCCTGCTCCTT	gB_76_GCN4_rev	CCCGCGGCGACGGTCGCGTTGTCGCCGGCGGGGCGCGGCGGCGGTGGGTTGCTGCCCACCAGCTTCTTCAGTCTGGCCACCTCGTTCTCCAGGTGGTAGTTCTTGGATCC
R-319	gB_95_galK_for	CGCCGCCGCGCCCCGCCGGCGACAACGCGACCGTCGCCGCGGGCCACGCCCCTGTTGACAATTAATCATCGGCA	gB_95_GCN4_for	CGCCGCCGCGCCCCGCCGGCGACAACGCGACCGTCGCCGCGGGCCACGCCGGATCCAAGAACTACCACCTGGAGAACGAGGTGGCCAGACTGAAGAAGCTGGTGGGCAGC
	gB_95_galK_rev	GTTTGCATCGGTGTTCTCCGCCTTGATGTCCCGCAGGTGCTCGCGCAGGGTTCAGCACTGTCCTGCTCCTT	gB_95_GCN4_rev	GTTTGCATCGGTGTTCTCCGCCTTGATGTCCCGCAGGTGCTCGCGCAGGGTGCTGCCCACCAGCTTCTTCAGTCTGGCCACCTCGTTCTCCAGGTGGTAGTTCTTGGATCC

**TABLE 3:** Oligonucleotides employed for the diagnostic PCR of the recombinant genomes (R-313, R-315, R-317, and R-319)

GalK recombination		GCN4 recombination	
Primer	Sequence	Primer	Sequence
galK_129_f	ACAATCTCTGTTTGCCAACGCATTTGG	gB_ext_for	GAGCGCCCCCGACGGCTGTATCG
galK_417_r	CATTGCCGCTGATCACCATGTCCACGC	gB_431_rev	TTGAAGACCACCGCGATGCCCT

To reconstitute the recombinant viruses R-313, R-315, R-317, R-319, and R-321, 500 ng of recombinant BAC DNA was transfected in SK-OV-3 cells by means of Lipofectamine 2000 (Life Technologies). Virus growth was monitored via green fluorescence. The recombinant viruses that encode the GCN4 peptide were reconstituted initially in SK-OV-3, frozen/thawed to lyse the SK-OV-3 cells, and subsequently grown in Vero-GCN4R cells. Virus stocks were generated in Vero-GCN4R and titrated in Vero-GCN4R, wt Vero, and SK-OV-3 cells. The sequence of the gB open reading frame (ORF) was verified by sequencing for each recombinant.

### Tropism of the recombinant viruses.

The indicated J cell derivatives, wt Vero, Vero-GCN4R, and SK-OV-3 cells were infected with R-313, R-315, R-317, R-319, and R-321 at an input multiplicity of infection (MOI) of 3 PFU/cell for 90 min at 37°C. The parental R-LM113 virus was included as a control. Pictures were taken 24 h after infection by a Nikon Eclipse TS100 fluorescence microscope. Where indicated, infection was carried out in the presence of monoclonal antibody (MAb) to HER2 (trastuzumab) (28 μg/ml).

### Determination of virus growth.

Vero-GCN4R and SK-OV-3 cells were infected at an input multiplicity of infection of 0.1 PFU/cell (as titrated in the correspondent cell line) for 90 min at 37°C; unabsorbed virus was inactivated by means of an acidic wash (40 mM citric acid, 10 mM KCl, 135 mM NaCl, pH 3). Replicate cultures were frozen at the indicated times (24 and 48 h) after infection, and the progeny was titrated in SK-OV-3. Results are expressed as the mean findings from three independent replicates ± standard deviations (SD).

### Cell viability assay.

SK-OV-3 and Vero-GCN4R cells were seeded in 96-well plates at 8 × 10^3^ cells/well and infected with the indicated viruses or mock infected for 90 min at 37°C. The input multiplicity of infection (as titrated in the correspondent cell line) was 3 PFU/cell in Vero-GCN4R and 10 PFU/cell in SK-OV-3 cells. alamarBlue dye (Life Technologies) was added to the culture medium (10 μl/well) at the indicated times after infection. The plates were incubated for 4 h at 37°C and read at 560 and 600 nm with the GloMax Discover system (Promega) to detect the reduced and oxidized forms, respectively, of alamarBlue dye. For each time point, cell viability was expressed as the percentage of alamarBlue reduction in infected versus uninfected cells, after subtraction of the background value (medium alone). Each point represents the average from at least three triplicate samples ± SD.

### Plating efficiency and relative plaque size.

Replicate aliquots of R-313, R-315, R-317, R-319, R-321, R-213, R-LM5, and R-LM113, containing the same amount of virus (50 PFU, as titrated in SK-OV-3 cells), were plated on Vero-GCN4R and SK-OV-3 monolayers. The infected monolayers were overlaid with medium containing agar, and the number of plaques was scored 3 days later. For plaque size determination, 10-fold dilutions of R-313, R-315, R-317, R-319, R-321, R-213, R-LM5, and R-LM113 were plated onto Vero-GCN4R and SK-OV-3 monolayers. The infected monolayers were overlaid with medium containing agar. Three days later, pictures of 6 plaques were taken at the fluorescence microscope for each virus. Plaque areas (pxE2) were measured with Nis Elements-Imaging Software (Nikon). Each result represents the average area ± SD.

### *In vivo* antitumor efficacy.

C57BL/6 mice transgenic for and tolerant to hHER2 [B6.Cg-Pds5bTg(Wap-ERBB2)229Wzw/J], received from Jackson Laboratories, were bred in the animal facility of the Department of Veterinary Medical Sciences, University of Bologna. They were implanted with the murine Lewis lung carcinoma 1 (LLC-1) cells made transgenic for hHER2 (hHER2-LLC-1), 0.2 × 10E6 cells/mouse (Leoni et al., unpublished). Three days later, mice received R-317 or R-LM113 and R-87 as control viruses, intratumorally (i.t.), in four dosages/mouse (1 × 10E8 PFU/injection) at 3 to 4 days' intervals. Each treatment group consisted of 5 mice. The tumor size was measured by means of a caliper at the indicated days, as described previously (23). Animal experiments were performed according to European directive 2010/63/UE, Italian laws 116/92 and 26/2014. The experimental protocols were reviewed and approved by the University of Bologna Animal Care and Use Committee (“Comitato per il Benessere degli Animali” [COBA]) and approved by the Italian Ministry of Health, Authorization # 86/2017-PR to Anna Zaghini.
